# Clinical Investigation of Periodontal Health in Smoker and Nonsmoker Individuals

**DOI:** 10.1155/ijod/7805251

**Published:** 2026-06-02

**Authors:** Yara G. Oweis, Suzan A. Al Bdour, Islam M. Abd-Raheam, Alaa H. A. Sabrah, Noor H. Ismail, Najla S. Kasabreh, Hearos A. Bedros, Mamoun A. Ahram, Nadia S. Ereifej

**Affiliations:** ^1^ Department of Restorative Dentistry, School of Dentistry, University of Jordan, Amman, Jordan, ju.edu.jo; ^2^ Department of Physiology and Biochemistry, School of Medicine, University of Jordan, Amman, Jordan, ju.edu.jo; ^3^ Department of Oral and Maxillofacial Surgery, Oral Medicine and Periodontology, School of Dentistry, University of Jordan, Amman, Jordan, ju.edu.jo; ^4^ Department of Prosthodontics, School of Dentistry, University of Jordan, Amman, Jordan, ju.edu.jo

**Keywords:** IL-1β, MMP-8, periodontal health, smoking, vaping

## Abstract

**Background:**

Vaping, argileh, and electronic cigarette (e‐cigarette) smoking are becoming popular among adolescents and conventional smokers. While the impact of conventional smoking on oral tissues is well‐established, the impact of other types of smoking on oral health is still unknown. This cross‐sectional study aimed to compare pro‐inflammatory cytokine levels, especially interleukin (IL)‐1β and matrix metalloproteinase (MMP)‐8 in the gingival crevicular fluid (GCF) between nonsmokers and different types of smokers. The plaque index (PI), bleeding on probing (BOP), and probing depth (PD) were also examined.

**Methods:**

Demographic data were collected from 112 participants attending the Jordan University Hospital using a questionnaire. The groups included were nonsmokers and smokers of either cigarettes, argileh, vaping, or a combination of different types. Besides the basic clinical examination, pro‐inflammatory cytokine levels were measured using ELISA.

**Results:**

The mean PI was significantly lower in nonsmokers compared to the combination smoking group and to all smoking groups combined (*p*  < 0.05). BOP was significantly higher in argileh smokers compared to the vaping group. No statistically significant differences were observed in MMP‐8 and IL‐1β levels among the studied groups.

**Conclusions:**

Within the limitations of this study, smoking status was not associated with significant differences in GCF levels of IL‐1β and MMP‐8 among periodontally healthy and gingivitis participants. However, differences in BOP were observed between specific smoking groups, suggesting that smoking modality may influence clinical inflammatory presentation.

## 1. Introduction

Periodontal status is commonly assessed using clinical parameters such as plaque index (PI), probing depth (PD), gingival index, bleeding on probing (BOP), and radiographic evaluation where indicated. Moreover, gingival crevicular fluid (GCF) quantity and composition can provide valuable insights into early inflammatory changes [1].

GCF is produced in very small amounts by a healthy periodontium and is secreted from the gingiva into the gingival crevices. Its composition is largely similar to blood plasma [2]. It is composed of electrolytes, peptides, bacteria, enzymes, organic molecules, such as albumins, globulins, lipoproteins, and fibrinogen, as well as various cellular components [3]. The diagnostic importance of GCF was recognized decades ago and has since been used as a noninvasive diagnostic tool for detecting periodontal disease [2]. Moreover, the concentration of its various components has been used to evaluate the changes in periodontal health before and after conventional periodontal therapy [4]. Individuals with active gingival disease have an increased volume of GCF partly due to increased vascular permeability, and changes in its composition such as increased protein concentration which can serve as indicators of disease severity and treatment outcomes [2, 3]. Biochemical analysis of GCF provides a valuable insight into the host immune response, with cytokines serving as key markers of inflammatory activity. Among these, interleukin (IL)‐1β, MMPs, and tumor necrosis factor (TNF)‐α which play a key role in the pathogenesis of periodontal disease [1, 5].

IL‐1β is produced by monocytes, macrophages, fibroblasts, and bone cells and is associated with inflammatory processes and tissue destruction. MMPs comprise a family of 23 proteolytic enzymes, among which matrix metalloproteinase (MMP)‐8 is the most active and plays a key role in both physiological and pathological breakdown of connective tissue at the sites of inflammation [5]. The activities of most MMPs are low in a normal and healthy periodontium accordingly, their detection in healthy periodontal tissues is difficult, whereas more MMP concentrations can be detected in diseased and inflammatory conditions of periodontal tissues [2], making it a useful biomarker for assessing periodontal inflammation [6]. It was also reported that IL‐1β concentrations significantly increase in periodontitis patients compared to healthy individuals which indicates the suitability of its use as a biomarker for periodontal inflammation [1]. Due to these properties, both biomarkers have been widely investigated as noninvasive and objective indicators of periodontal disease activity evaluating treatment outcomes and identifying individuals at risk using GCF [1].

Tobacco smoking accounts for approximately 5.4 million deaths per year worldwide [7]. Its adverse effects on oral tissues are well established. Tobacco smoking reduces blood flow to gingival tissues and is associated with conditions such as aphthous stomatitis and oral squamous cell carcinoma [8, 9]. Smoking is also known to impair the immune response leading to a decrease in polymorphonuclear leukocytes in GCF which play a key role in the elimination of periodontal pathogens. This contributes to an increase in the extent and severity of periodontal destruction [10–12]. Furthermore, nicotine has been linked to an increased expression of destructive inflammatory cytokines, such as IL‐1β, IL‐6, and TNF‐α, and MMP‐8 in the GCF which further aggravates periodontal inflammation [9].

Electronic cigarettes (e‐cigarettes) have become commercially available in 2006 [4] and have been marketed as a healthier alternative to conventional cigarettes [7, 13]. Studies have reported that e‐cigarette smokers may experience oral manifestations such as dry mouth, burning sensations, altered taste, and halitosis [14]. Moreover, e‐cigarette smokers tend to exhibit increased PI, PD, bone loss, and GCF with higher concentrations of inflammatory markers compared to nonsmokers [15].

Argileh (waterpipe) smoking has become very popular not only in the Middle East, but also worldwide due to its social acceptability and the availability in multiple flavors [16–18]. Argileh smoking poses several health risks; a single argileh session has been reported to raise blood nicotine levels to amounts comparable to those observed with smoking multiple cigarettes besides many other known health hazards [18]. It was also reported that argileh smoking is associated with increased risk of periodontal bone loss and dry socket after tooth extractions [19].

To date, research investigating the effect of different types of smoking on pro‐inflammatory cytokines in GCF among healthy individuals remains scarce. Table S1 summarizes selected studies that have investigated pro‐inflammatory cytokine concentrations such as IL‐1β and MMP‐8 in healthy and periodontitis smoker and nonsmoker individuals. Despite these contributions, a gap persists in understanding how different types of smoking, such as conventional cigarette smoking, argileh, vaping, and combined smoking habits may influence pro‐inflammatory cytokine levels in GCF.

The null hypotheses were: (1) No statistically significant differences exist in GCF levels of MMP‐8 and IL‐1β among cigarette smokers, argileh smokers, vape users, combination smokers, and nonsmokers and (2) No statistically significant differences exist in clinical periodontal parameters (PI and BOP) among the same groups. Accordingly, the objective of this study was to compare PI and BOP, as well as MMP‐8 and IL‐1β levels in GCF, among cigarette smokers, argileh smokers, vape users, individuals practicing a combination of these smoking methods, and nonsmokers.

## 2. Methods

### 2.1. Demographic Data and Sample Selection

All information and data collected in this cross‐sectional study were performed during the everyday standard of care for patients. No invasive procedures were involved. The study was approved by the research committees of the Institutional Review Board of the Deanship of Scientific Research at the University of Jordan (10/2020/21608) and complied with the Declaration of Helsinki.

Prior to enrollment into the study, all participants were informed about the purpose and protocol of the study. A verbal informed consent was taken from the participants in the presence of a dental assistant, who documented the consent on the participants’ examination sheets. Written informed consent was not required, as the procedure involved a noninvasive technique associated with minimal risk; this approach was approved by the institutional review board.

Demographic data, including age, gender, and daily frequency and duration of smoking and vaping were collected from 112 participants attending the Jordan University Hospital between January 2021 and December 2021 with no underlying systemic disease. The initial target sample size was 150 participants (30 per group across five groups). However, due to recruitment limitations related to strict inclusion criteria and the uneven distribution of smoking habits in the source population, this number could not be achieved.

Participants were classified into five groups: nonsmokers, cigarette smokers, argileh smokers, vape users, and combination smokers, based on the Centers for Disease Control and Prevention (CDC) criteria. Cigarette smokers were those who had smoked ≥5 cigarettes daily for at least 1 year. Vape users were those who had used vape exclusively at least once daily. Argileh smokers were those who had smoked argileh at least once a week for more than 1 year. Combination smokers were those who had used more than one of the previously mentioned methods. Nonsmokers were those who had never used any form of tobacco products. Exclusion criteria included alcohol consumption; the presence of systemic diseases; use of systemic medications; current or previous periodontal treatment; clinically diagnosed periodontitis; and more than four missing teeth (excluding third molars). Periodontitis was excluded based on clinical assessment, including PD > 4 mm, and the presence of clinical attachment loss, as defined by 2017 classification [20]. Radiographic assessment was not performed.

### 2.2. Clinical Examination

All clinical measurements were performed by a single, trained examiner following standardized protocols. Although formal calibration data were not collected prior to the study, the examiner’s training and adherence to consistent measurement techniques were intended to minimize variability. The number of missing teeth was recorded. Full‐mouth PI, BOP, and PD were measured for all participants on the mesio‐buccal, mid‐buccal, disto‐buccal, distolingual, mid‐lingual, and mesio‐lingual surfaces of all maxillary and mandibular teeth [9]. CAL was assessed during screening to exclude individuals with any evidence of attachment loss; following exclusion of such individuals, CAL was not recorded as an outcome measure in the study, as all remaining participants were periodontally healthy. Baseline cleaning of teeth was performed for all participants. They were given oral hygiene instructions and were dismissed for the second visit, which was 1 month later.

During the second visit, GCF collection was done using a sterile paper strip (Periopaper, Amityville, NY, USA). To ensure accuracy and reproducibility, all GCF samples were collected by the same examiner using standardized techniques. Before GCF collection, the site was isolated with cotton rolls, the area was dried using triple syringe, and the sterile paper‐strip was inserted into the gingival crevice until resistance was felt. It was held in place for 30 s. Samples were collected from three different sites for each patient (the mid‐buccal aspect of the mandibular right and left first molars and the mid‐buccal aspect of one of the maxillary first molars). Samples contaminated with blood were discarded. Sample handling followed a strict protocol to preserve analyte stability. All samples were processed immediately after collection as follows, the strips were placed in labeled sterile 1.5 mL Eppendorf tubes containing 300 μL of 0.01M phosphate buffered saline (pH 7.2) and protease‐inhibitor (protease inhibitor cocktail 50×, Promega, Madison, USA). The tubes were left for 45 min then centrifuged for 5 min at 13,000 rpm at 4°C. The supernatant was collected and stored at −80°C for further analysis. All GCF samples were analyzed for pro‐inflammatory cytokine levels within 1 week of collection using standardized ELISA protocols to minimize biomarker degradation and ensure reliable quantification. The experimental assays were done at the Cellular Biochemistry Research Laboratory, School of Medicine, The University of Jordan.

### 2.3. Human IL‐1β and Human Total MMP‐8

IL‐1β and total MMP‐8 were measured using Quantikine ELISA enzyme‐linked immunosorbent assay plate (R&D Systems, Minneapolis, USA) in duplicates according to the manufacturer’s instructions. The concentrations of IL‐1β in the GCF samples were calculated based on a seven‐point standard curve (2.5−3.9 pg/mL) using two‐fold serial dilutions of recombinant human IL‐1β standard provided with the kit (Part No. 890041). Polyclonal antibody specific for human IL‐1β (Part No. 890040; provided with the kit) was used to measure natural and recombinant human IL‐1β. The concentration of the reaction end‐product was measured spectrophotometrically at 450 nm. A standard curve was constructed by plotting the mean absorbance for each standard on the *y*‐axis against the concentration on the *x*‐axis and drawing a best curve through the points on the graph. The data were linearized by plotting the log of the human IL‐1β concentrations versus the log of the optical density (OD) and the best fit line can be determined by regression analysis. Considering specificity of the kit provided, the following factors below were prepared at 50 ng/mL in calibrator diluent and assayed for cross‐reactivity. Preparations of the following factors at 100 ng/mL in a mid‐range recombinant human IL‐1β control were assayed for interference. No significant cross‐reactivity or interference was observed with the following: recombinant human: IL‐1 α, IL‐1F7, IL‐1ra, IL‐1 RAcP, IL‐1 RAPL1, IL‐1 RAPL2, IL‐1 Rrp2, IL‐18, IL‐18 Rα, IL‐18 Rβ, IL‐33, IL‐36 α, IL‐36β, IL‐36γ, IL‐36ra, IL‐38 SIGIRR, and ST2. Recombinant human IL‐1 RI and IL‐1RII do not cross‐react but does interfere at concentrations higher than 10,000 pg/mL.

The concentrations of human total MMP‐8 in the GCF samples were calculated based on a seven‐point standard curve (10−0.156 ng/mL) using two‐fold serial dilutions of recombinant human MMP‐8 standard (Part No. 899009, provided with the kit). Monoclonal antibody specific for human MMP‐8 was used to determine the natural and recombinant human MMP‐8 (Part No. 899008, provided with the kit). The samples were diluted 10‐fold with Calibrator Diluent RD5P (diluted 1 : 5).

Thirty microliters of the sample were added to 270 µL Calibrator Diluent RD5P (Part No. 895151 provided with the kit). A standard curve was created by plotting the mean absorbance for each standard on the *y*‐axis against the concentration on the *x*‐axis and drawing a best curve through the points on the graph. The data were linearized by plotting the log of human MMP‐8 concentrations versus the log of the OD and the best fit line can be determined by regression analysis. The concentration of the reaction product was measured spectrophotometrically at 450 nm. The concentration readings from the standard curve were multiplied by the dilution factor.

Considering specificity of the kit provided, the following factors prepared at 100 ng/mL in calibrator diluent and assayed for cross‐reactivity. Preparations of the following factors prepared at 100 ng/mL in a mid‐range recombinant human MMP‐8 control were assayed for interference. No significant cross‐reactivity or interference was observed. Recombinant human: ADAM8, ADAM9, ADAM10, ADAM12, ADAM15, ADAM19, ADAM22, ADAM23, ADAM28, ADAM33, ADAMTS1, ADAMTS4, ADAMTS5, ADAMTS13, ADAMTS15, Lipocalin‐2, MMP‐1, MMP‐2, MMP‐3, MMP‐7, MMP‐9, MMP‐10, MMP‐12, MMP‐13, TACE, TIMP‐1, TIMP‐2, TIMP‐3, and TIMP‐4.

A microplate reader capable of measuring absorbance at 450 nm, with the correction wavelength set at 540 or 570 nm was used to measure human IL‐1β and human total MMP‐8. (Synergy HTX multi‐mode microplate reader, BioTek Instruments Inc., Vermont, USA) [21].

### 2.4. Statistical Analysis

Statistical analysis was performed using SPSS software (Version 23 for Windows, IBM, Chicago, IL., USA). Data were tested for normality using the Shapiro Wilk test. Most of the parameters were not normally distributed, accordingly, Kruskal–Wallis analysis of variance followed by Dunn’s post hoc test were used for pairwise comparisons. Categorical variables were compared between groups using the Chi‐square test. Pearson’s Correlation coefficient was used to evaluate correlations between biomarkers, clinical parameters, and smoking duration. Linear regression analysis was performed to evaluate the effect of smoking duration on cytokine levels while controlling for potential confounders. A *p*‐value <0.05 was considered statistically significant. The power analysis was performed using IL‐1β levels as the primary outcome variable and smoking status as the independent variable. With a sample size of 112, an alpha level of 0.05, and an observed effect size of 0.08, the study achieved a statistical power of 64%. No missing data were identified.

## 3. Results

### 3.1. Study Population and Demographics

A total of 112 participants were included in the study with 56% were males and 44% were females. The age ranged between 18 and 49 with a mean age of 26 years old. All examined participants had a PD of ≤4 mm and were classified as healthy or gingivitis participants according to the periodontal classification of 2017 [20].

Of the 112 participants, 31 were nonsmokers, 19 were cigarette smokers, 18 were argileh smokers, 20 smoked vape, and 24 were combination smokers. A significant association was found between gender and smoking status (Pearson Chi‐square = 38.08, df = 4, *p*  < 0.001) (Table 1). Among males, the most common smoking pattern was combined smoking (31.7%), followed by vaping (28.6%), cigarette smoking (19.0%), and argileh smoking (12.7%), while only 7.9% were nonsmokers. In contrast, more than half of the examined females were nonsmokers (53.1%), followed by argileh smoking (20.4%), cigarette smoking (14.3%), combination smoking (8.2%), and vaping (4.1%). Table 1 displays the distribution of different types of smoking among male and female individuals.

**Table 1:** The distribution of different types of smoking according to gender is presented as percentages within each gender group.

Smoking type	Nonsmoker (*n* = 31) *n* (%)	Cigarette (*n* = 19) *n* (%)	Argileh (*n* = 18) *n* (%)	Vape (*n* = 20) *n* (%)	Combination (*n* = 24) *n* (%)
Male (*n* = 63)	5 (7.9%)	12 (19.0%)	8 (12.7%)	18 (28.6%)	20 (31.7%)
Females (*n* = 49)	26 (53.1%)	7 (14.3%)	10 (20.4%)	2 (4.1%)	4 (8.2%)
Total	31 (27.7%)	19 (17.0%)	18 (16.1%)	20 (17.9%)	24 (21.4%)

*Note:* Comparisons between males and females were performed using the chi‐square test. Pearson chi square: 38.75, df = 4, *p*  < 0.001.

### 3.2. Biomarkers and Clinical Parameters Comparisons Across Groups

The mean and the standard deviation for the concentration of pro‐inflammatory cytokines (MMP‐8, and IL‐1β) as well as that for PI and BOP are presented in Table 2. Since participants with PD > 4 mm were excluded, all enrolled individuals presented with healthy PDs. Given the resulting minimal variation across groups, PD data are not reported in Table 2. Kruskal–Wallis analysis of variance was used to compare all four outcome variables across the five smoking groups, since most variables were not normally distributed. The mean MMP‐8 concentration was highest in cigarette smokers (7.9 ± 10.0 ng/mL) with no statistically significant difference between groups. Similarly, IL‐1β levels were the highest in argileh smokers (0.23 ± 0.16 ng/mL) yet no statistically significant difference was found between groups. In contrast, PI differed significantly across groups (*p* = 0.006). Post hoc Dunn’s pairwise comparisons with Bonferroni correction revealed that the mean PI was significantly lower in nonsmokers compared with the combination smoking group (*p* = 0.02), with all other pairwise comparisons being nonsignificant.

**Table 2:** The mean and the standard deviation of the concentration of pro‐inflammatory cytokines (MMP‐8 and IL‐1β) (ng/mL), PI, and BOP by smoking group.

Measured parameters	Nonsmokers (*n* = 31), mean ± SD	Smokers (*n* = 81)			
		Cigarette (*n* = 19), mean ± SD	Argileh (*n* = 18), mean ± SD	Vape (*n* = 20), mean ± SD	Combination (*n* = 24), mean ± SD
MMP‐8	4.8 ± 4.2	7.9 ± 10	6.1 ± 6.0	4.2 ± 3.6	5.2 ± 3.8
IL‐1β	0.13 ± 0.08	0.19 ± 0.13	0.23 ± 0.16	0.16 ± 0.12	0.16 ± 0.91
PI	0.35 ± 0.5^a^	0.72 ± 0.8	0.80 ± 0.70	0.80 ± 0.2	0.71 ± 0.5^a^
BOP	6.4 ± 7.0	5.2 ± 4.8	11.0 ± 8.0^a^	2.9 ± 4.8^a^	5.4 ± 5.0

^a^Indicates a statistically significant difference between the two groups.

BOP also differed significantly across groups (*p* = 0.02). The lowest BOP was recorded in the vape group (2.9% ± 4.8%) and the highest in the argileh group (11.0% ± 8.0%). A statistically significant difference was found between the vape and argileh groups (*p* = 0.018) (Table 2).

To provide a broader comparison of clinical parameters and inflammatory cytokine levels, all smokers (*n* = 81) were pooled and compared against nonsmokers (*n* = 31) using Mann–Whitney *U* test. Results are presented in Table 3. MMP‐8 levels did not differ significantly between smokers (mean = 5.8 ± 6.2 ng/mL) and nonsmokers (4.8 ± 4.2 ng/mL). IL‐1β levels were numerically higher in smokers (0.18 ± 0.12 ng/mL) compared to nonsmokers (0.13 ± 0.08 ng/mL), approaching but not reaching statistical significance (*p* = 0.051). PI was significantly higher in smokers with a mean of 0.76 ± 1.2 when compared to nonsmokers with a mean of 0.35 ± 0.56 (*p* = 0.02), whereas no significant difference was found in the mean BOP between smokers and nonsmokers (6.0 ± 6.5 and 6.5 ± 7.8, respectively) as seen in Table 3. These findings suggest a trend toward elevated IL‐1β and PI in smokers.

**Table 3:** The mean and the standard deviation of the concentration of pro‐inflammatory cytokines (MMP‐8 and IL‐1β) (ng/mL), PI, and BOP between smokers and nonsmokers.

Variable	Smokers (*n* = 81), mean ± SD	Nonsmokers (*n* = 31), mean ± SD	*p*‐Value
MMP‐8	5.8 ± 6.2	4.8 ± 4.2	0.56
IL‐1β	0.18 ± 0.12	0.13 ± 0.08	0.051
PI	0.76 ± 1.2	0.35 ± 0.56	0.02^a^
BOP	6.0 ± 6.5	6.5 ± 7.8	0.80

^a^Indicates a statistically significant difference (*p*  < 0.05).

### 3.3. Correlation Analysis

Pearson’s correlation coefficients between pro‐inflammatory cytokine levels (IL‐1β and MMP‐8), clinical parameters (PI and BOP), and smoking duration are presented in Table 4. Both MMP‐8 and IL‐1β demonstrated weak, nonsignificant association with PI (*r* = 0.05, *p* = 0.61 for both) and BOP (*r* = 0.02, *p* = 0.84; *r* = 0.10, *p* = 0.20) for MMP‐8 and IL‐1β, respectively. MMP‐8 showed a weak, nonsignificant correlation with smoking duration (*r* = 0.12, *p* = 0.20). In contrast, IL‐1β demonstrated a weak but statistically significant positive correlation with smoking duration (*r* = 0.23, *p* = 0.02), indicating that longer smoking duration was associated with higher IL‐1β levels in GCF.

**Table 4:** Correlation between biomarker concentrations and clinical findings.

Variable	*r*	*p*‐Value
MMP‐8		
PI	0.05	0.61
BOP	0.02	0.84
Duration	0.12	0.20
IL‐1β		
PI	0.05	0.61
BOP	0.10	0.20
Duration	0.23	0.02^a^
Duration		
PI	0.02	0.90
BOP	0.22	0.02^a^

^a^Indicates a statistically significant difference.

Regarding clinical parameters, PI showed a weak, nonsignificant correlation with smoking duration (*r* = 0.02, *p* = 0.90), while BOP demonstrated a weak but statistically significant positive correlation with smoking duration (*r* = 0.22, *p* = 0.02), as shown in Table 4.

### 3.4. Linear Regression

Linear regression analysis was performed to evaluate the independent effect of smoking duration on cytokine levels (MMP‐8 and IL‐1β), while controlling for potential confounding factors. Smoking duration was significantly associated with IL‐1β levels (*B* = 5.16, *R* ^2^ = 0.04, *p* = 0.04), indicating that each additional year of smoking was associated with a 5.16 ng/mL increase in GCF IL‐1β levels. In contrast, smoking duration was not significantly associated with MMP‐8 levels (*B* = 0.17, *R* ^2^ = 0.02, *p* = 0.17), as presented in Table 5.

**Table 5:** Linear regression results of the association between smoking duration and pro‐inflammatory cytokine levels.

Smoking duration	Value
MMP‐8	
*B*	0.17
*R* ^2^	0.02
*p*‐Value	0.17
IL‐1β	
*B*	5.16
*R* ^2^	0.04
*p*‐Value	0.04 ^∗^

^∗^ *p* < 0.05.

## 4. Discussion

In this cross‐sectional study, clinical periodontal parameters (PI and BOP) and pro‐inflammatory cytokine levels in GCF were compared among cigarette smokers, vape users, argileh smokers, individuals with combined smoking habits, and nonsmokers. The findings of this study suggest that, in this study population, smoking did not have a measurable impact on pro‐inflammatory cytokine levels despite increased plaque accumulation.

The results of the present study demonstrated no statistically significant differences in MMP‐8 and IL‐1β levels in the GCF between nonsmokers and smoker participants. This finding is in agreement with a recent study that reported no significant effect of smoking on the levels of MMP‐8 and IL‐1β in the GCF of periodontally healthy smokers [22]. In contrast, studies that have reported elevated levels of pro‐inflammatory cytokine levels among smokers often included patients with periodontitis, in which inflammation and tissue destruction were more pronounce and partially attributed to the presence of existing disease [9, 23, 24]. This may partly explain the differences noted with previous studies since both IL‐1β and MMP‐8 are associated with disease severity rather than early or subclinical inflammatory changes [1]. Moreover, other smoking types such as argileh, e‐cigarettes, vape, and combined smoking may vary in their biological effects. For example, some studies have reported higher cytokine levels in cigarette smokers compared to nonsmokers, while no significant differences were observed between e‐cigarette users and nonsmokers [9, 23].

Advanced periodontal disease is known to affect cytokine levels making it difficult to isolate the effect of smoking. Restricting the sample helps ensure that observed differences are more likely related to smoking exposure rather than disease severity. This allows for a more accurate comparison of cytokine levels between groups. While the comparison between the different studied groups was not significant, IL‐1β demonstrated a significant positive correlation with smoking duration (*r* = 0.23, *p* = 0.02), which was further supported by linear regression analysis (*p* = 0.04), suggesting that cumulative smoking exposure may gradually elevate IL‐1β levels in GCF even in periodontally healthy individuals. This finding highlights the potential value of longitudinal studies to detect dose‐dependent inflammatory changes.

Several factors related to the study methodology and target population may have influenced the findings of the present study. It was previously reported that variations in GCF collection methods, including differences in sample volume and the number of paper strips used, as well as differences in ELISA kits and their detection sensitivities, may affect the accuracy and comparability of biomarker measurements [1]. Furthermore, an observed statistical power of 64% at a small effect size of 0.08 may have been insufficient to reliably detect small differences between groups. Moreover, potential systemic factors such as psychological stress may also influence pro‐inflammatory cytokine levels in GCF and consequently affect the periodontal inflammatory response [5]. However, in periodontally healthy individuals, where inflammatory activity is minimal, the levels of these biomarkers may remain relatively stable, which may explain the lack of significant differences observed in the present study.

Although GCF sampling is considered minimally invasive, it is technically demanding and several factors may affect measurement accuracy. The limited sample volume, the risk of contamination with saliva or blood, variations in sulcus depth, the presence of gingival inflammation, and patient movement during sample collection may influence the reliability of the measurements [25]. To minimize variability, a standardized protocol was implemented to ensure accurate and reproducible sample collection. This included careful isolation of the target site, gentle insertion of sterile Perio paper strips, exclusion of contaminated samples, and collection by a single trained examiner. Immediate processing and low‐temperature storage were employed to preserve biomarker stability, minimize pre‐analytical variability and keep cytokine measurements accurate.

Our results showed that nonsmokers had a significantly lower mean PI compared to the combination smoking group, with no statistically significant differences observed between nonsmokers and other individual smoking groups. When smokers were grouped together and compared to nonsmokers, nonsmokers had a significantly lower PI than smokers. This is in agreement with previous studies which reported that smokers had higher PI compared to nonsmokers [9, 23, 24]. Dental plaque develops following the formation of an acquired pellicle on the tooth surface, which is then colonized and modified by bacterial activity [26]. Smoking has been shown to change the subgingival microbial environment, favoring the growth of pathogenic species that can trigger inflammatory response [27–29]. Even clinically healthy smokers may possess a highly diverse, and pathogenic anaerobic microbiome, placing them at increased risk for periodontal damage [29]. Consequently, the higher PI observed among smokers may be attributed to a combination of behavioral and biological factors, including poorer oral hygiene practices, reduced salivary flow [30], and smoking‐induced changes in the oral microbiome [27–29]. Moreover, nicotine‐related vasoconstriction and impaired immune response may facilitate plaque accumulation [31].

BOP is a vital component of comprehensive oral examination and is widely used as a clinical indicator of periodontal inflammation. Although it may give false positive results, the absence of bleeding is generally considered a reliable sign of periodontal health due to its high specificity despite low sensitivity for disease progression [32, 33]. Our results showed that the mean BOP values were comparable across all groups except for the argileh and vape smoker groups with vape smokers recording the lowest mean BOP values and argileh smokers the highest (*p* = 0.018). No significant differences were observed between nonsmokers and any other smoking groups. Previous studies have reported smokers have less or delayed gingival bleeding when compared to nonsmokers [31, 34]. Several mechanisms have been proposed to explain this effect, including gingival vasoconstriction, decreased angiogenesis, thermally induced nerve damage, changed microbiota, and changed immune response, however, the exact mechanism remains incompletely understood [31]. In the present study, the lack of significant difference between cigarette smokers and nonsmokers may be related to participants’ age, smoking type, and dose [31, 35, 36]. Our cohort consisted of participants aged between 18‐49 with a mean age of 26 years, reflecting a relatively young sample on average, with a likely lower cumulative smoking exposure. This may explain the absence of significant differences between nonsmokers and smokers. The increased BOP observed among argileh smokers may be attributed to both general and argileh‐specific mechanisms. Unlike other forms of smoking, argileh smoking involves heating tobacco with charcoal [19], producing additional toxic byproducts, particularly carbon monoxide (CO) [37]. At high concentrations, CO binds to hemoglobin, leading to tissue hypoxia and increased cellular stress, mucosal lesions, and microvascular fragility, which may contribute to increased bleeding susceptibility [38]. The low BOP values among vape smokers may be due to the vasoconstrictive effects of nicotine on the tissues, combined with the absence of combustion byproducts such as CO and other materials present in argileh and tobacco smoke. Moreover, nicotine‐induced vasoconstriction and the effect on the local immune response may suppress gingival vascularity and adversely affect the bleeding response [31, 34]. Additionally, variations in oral hygiene practices among the participants might have also influenced these findings, as inadequate plaque control is a well‐established contributor to gingival inflammation and bleeding. In general, these findings highlight the complex and multifactorial effect of different smoking modalities on periodontal tissues.

While additional parameters such as CAL and radiographic bone loss provide a more comprehensive assessment of periodontal status, they are more relevant in individuals with established periodontitis. In the present study, participants with any evidence of CAL or PD >4 mm were excluded to ensure the inclusion of periodontally healthy individuals; therefore, CAL was not applicable to this population, as it reflects cumulative previous tissue destruction rather than current inflammatory activity. The study, therefore, focused on parameters indicative of current periodontal condition and oral hygiene, including PI and BOP, which are more sensitive to ongoing inflammatory changes and better aligned with the study objectives of detecting early periodontal response within a periodontally healthy cohort. Similarly, although subgingival microbiota analysis could have further enhanced mechanistic interpretation, the combination of clinical and cytokine parameters was considered sufficient to address the study objectives. Future studies incorporating microbial profiling would strengthen the biological interpretation of smoking‐related periodontal effects.

Regarding sample size, the initial target of 150 participants (30 per group) could not be achieved due to strict inclusion criteria and the uneven distribution of smoking habits within the source population. All five study groups were ultimately represented, with a minimum of 18 participants per group. However, the achieved statistical power was 64%, falling below the conventionally accepted threshold of 80%, with an observed effect size of 0.08. This may have limited the ability to detect small differences between groups, particularly for pro‐inflammatory cytokine levels.

The complex interplay of host defense mechanisms and the diversity of smoking products make it difficult to draw definitive conclusions about the different effects of smoking modalities on GCF composition and periodontal health, however, the present findings suggest that these effects may vary according to smoking type and host‐related factors.

Future research should aim at comparing the effect of different smoking modalities in individuals at varying stages of periodontal disease, using larger multicenter cohorts to improve the generalizability of findings. Moreover, expanding the sample size will also be essential for achieving adequate statistical power, particularly when examining cytokine‐level outcomes. From a clinical standpoint, these findings emphasize the importance of good oral hygiene practice and regular professional monitoring to mitigate the cumulative effects of smoking on gingival tissues.

Future research should explore strategies to enhance oral health behaviors, including the incorporation of medical coaching approaches. Additionally, artificial intelligence‐based technologies show promise in improving the prediction and evaluation of periodontal biomarkers and supporting clinical decision‐making. However, their integration into practice should be undertaken with careful consideration of ethical and regulatory challenges, including data security, transparency, and clinical accountability, as highlighted by D’Albis and Capodiferro [39].

## 5. Limitations

This study has several limitations that should be acknowledged. The relatively young sample and unequal group sizes may have limited the detection of cumulative smoking effects and reduced between‐group comparability. Additionally, measuring inflammatory markers at only one point in time does not show how cytokine levels change over time. Moreover, the absence of subgingival microbiota profiling precludes a full mechanistic interpretation of the observed periodontal changes. Finally, single‐center recruitment limits the generalizability of the findings.

## Author Contributions

Yara G. Oweis conceptualized the study and was responsible for formal analysis, investigation, resources, visualization, supervision, project administration, and funding acquisition, and had full access to all of the data in this study, taking complete responsibility for the integrity of the data and the accuracy of the analysis. Noor H. Ismail contributed to the methodology and participated in writing the original draft. Mamoun A. Ahram performed validation and contributed to reviewing and editing the manuscript. Hearos A. Bedros and Najla S. Kasabreh were responsible for data curation. Alaa H. A. Sabrah and Islam M. Abd‐Raheam contributed to writing the original draft. Nadia S. Ereifej participated in reviewing and editing the manuscript.

## Funding

This research was funded by the Deanship of Scientific Research at University of Jordan (Grant 1/2019/1815).

## Disclosure

All authors have read and approved the final version of the manuscript.

## Conflicts of Interest

The authors declare no conflicts of interest.

## Supporting Information

Additional supporting information can be found online in the Supporting Information section.

## Supporting information


**Supporting Information** The supporting information file provides a summary table of selected published studies that assessed IL‐1β and MMP‐8 levels in gingival crevicular fluid across different study groups, including healthy individuals and patients with gingivitis or periodontitis, in both smokers and nonsmokers.

## Data Availability

The data that support the findings of this study are available from the corresponding author upon reasonable request.
