# Supplementary material for: Clinical Investigation of Periodontal Health in Smoker and Nonsmoker Individuals
Source: Int J Dent. 2026 Jun 2;2026:7805251. doi: 10.1155/ijod/7805251 (PMC13239554; doi:10.1155/ijod/7805251)
Supplement: Supplementary file 1 — Supporting Information The supporting information file provides a summary table of selected published studies that assessed IL‐1β and MMP‐8 levels in gingival crevicular fluid across different study groups, including healthy individuals and patients with gingivitis or periodontitis, in both smokers and nonsmokers. [file IJOD-2026-7805251-s001.docx]

| Units | Main findings | Pro-inflammatory cytokine | Study |
| --- | --- | --- | --- |
| pg/mL | Cigarette smokers: 0.13 ±0.02  Electronic smokers: 0.06± 0.01  Non-smokers: 33.3 ± 11.4 | IL- 1β | BinShabaib M et al. 2019[9] |
| pg/mL | Cigarette smokers: 2.1 ± 0.2  Electronic smokers: 0.9 ± 0.1  Non-smokers: 0.7 ± 0.1 | MMP-8 | BinShabaib M et al. 2019[9] |
| ng | Gingivitis: 5.7 ±7.7  **Periodontitis**  Shallow pockets: 4.7 ±6.3  Deep pockets: 6.1 ±10.7 | MMP-8 | Figueredo CM et al. 2005[20] |
| pg  ng | Healthy individuals: 0.04 (0.02-0.07)  Healthy individuals: 10.4 (7.5- 15.9) | IL-1β  MMP-8 | Miranda LA et al. 2006[21] |
| µg/L | Healthy individuals: 1268 ± 2126  Smokers:3997 ± 3126 | MMP-8 | Mäntylä P et al. 2006[22] |
| ng/mL | Healthy individuals: 174.17± 22.40  Periodontitis  Smokers: 459.16± 24.30  Non-smokers: 354.83± 29.19 | MMP-8 | Gupta N et al. 2016 |
| pg/mL | Smokers: 44.336  Non-smokers:50.513 | MMP-8 | Do HT et al. 2023 |
| ng/mL | Periodontitis: 92.04 (72.19) | MMP-8 in | Keskin M. et al. 2023[23] |
| pg/μL | Healthy individuals: 2.3 ±1.46  Periodontitis patients: 4.16 ± 2.85 | IL-1β | Bascones-Martínez A et al. 2012 [24] |
| pg | Healthy individuals: 73  Gingivitis patients: 75  Periodontitis patients 90 |  | Afacan B et al. 2023[25] |

**Table S1.** Proinflammatory cytokine concentrations of IL-1β and MMP-8 across study groups healthy and periodontitis patients, smokers and non-smokers as reported in the literature
